# Supplementary material for: The Effect of JAK Inhibitor on the Survival, Anagen Re-Entry, and Hair Follicle Immune Privilege Restoration in Human Dermal Papilla Cells
Source: Int J Mol Sci. 2020 Jul 20;21(14):5137. doi: 10.3390/ijms21145137 (PMC7404120; doi:10.3390/ijms21145137)
Supplement: Supplementary file 1 [file ijms-21-05137-s001.pdf]

Supplementary Materials

The Effect of JAK Inhibitor on the Survival, Anagen Re-Entry, and Hair Follicle Immune Privilege Restoration in Human Dermal Papilla Cells

Table S1. Primer sequence and PCR conditions.

Species	Primer Name	Forward	Reverse
Human	*caspase-1*	TATCCGTTCCATGGGTGAAG	TCAAAGCTCGGGTCTTATCC
	IL-1b	GCCAATCTTCATTGCTCAAGT	ACTTCATCTGTTTAGGGCCA
	IL-15	GCCCAAAGCACCTAACCTAT	GTTCCCAATACCATGAAGGC
	IL-18	AGCTGAAGATGATGAAAACCT G	ATAGAGGCCGATTTCCTTGG
	DKK1	TGTGCTAGACACTTCTGGTC	TTCTCCACAGTAACAACGCT
	b-catenin	GTGTTAAGTTATAGTGAATACTGCT	GTGTTCTACACCATTACTCAATTCT
	Lef1	AGCTGCCTACATCTGAAACA	TGGAGACAGTCTGGGTTTTC
	WNT7a	TCTGTAACAAGATCCCAGGC	CATTGCGGAACTGAAACTGA
	TGF-b2	CTTTTCTGCGTCAGTGTGAG	ACTTTTCCTAGCCCAGTCAC
	FGF2	TCCACCTATAATTGGTCAAAGTGGT	CATCAGTTACCAGCTCCCCC
	FGF7	AATTGTGGCAATCAAAGGGG	CCGTTGTGTGTCCATTTAGC
	IGF1	AATCCCTCTTCTGCTTGCTA	ATTTTCCCCATCGCTTCTGA
	PDGF	TGAAGAGCAAAGTGATTTCTTACA	GGACAGCCTTTCGTAGAAGA
	VEGFa	AGAGGCTTGGGGCAGCCGAG	ACTCCCGGGCTGGTGAGTCC
	GAPDH	CCCCAGCAAGGACACTGAGCAA	GGCTCCCTAGGCCCCTCCTGTTAT
